# A reaction norm model for genomic selection using high-dimensional genomic and environmental data

**DOI:** 10.1007/s00122-013-2243-1

**Published:** 2013-12-12

**Authors:** Diego Jarquín, José Crossa, Xavier Lacaze, Philippe Du Cheyron, Joëlle Daucourt, Josiane Lorgeou, François Piraux, Laurent Guerreiro, Paulino Pérez, Mario Calus, Juan Burgueño, Gustavo de los Campos

**Affiliations:** 1 Department of Biostatistics, University of Alabama at Birmingham, 1665 University Boulevard, 327L Ryals Public Health Building, Birmingham, AL 35216 USA; 2 Present Address: Agronomy and Horticulture Department, University of Nebraska, 321 Keim Hall, Lincoln, NE USA 68583-0915; 3 Biometrics and Statistics Unit, International Maize and Wheat Improvement Center (CIMMYT), Apdo. Postal 6-641, 06600 Mexico, D.F., México; 4 Arvalis Institut du végétal, Station Inter-institut, 6 chemin de la côte vieille, 31450 Baziège, France; 5 Colegio de Postgraduados, Montecillo, Edo. de México, Mexico, México; 6 Arvalis Institut du végétal, IBP Université Paris Sud, Rue de Noetzlin, Bât. 630, 91405 Orsay, France; 7 Arvalis Institut du vegetal, Station expérimentale, 91720 Boigneville, France; 8 Arvalis Institut du végétal, 3 rue Joseph et Marie Hackin, 75116 Paris, France; 9 Animal Breeding and Genomics Centre, Wageningen UR Livestock Research, P.O. Box 135, 6700 AC Wageningen, The Netherlands

## Abstract

*****Key message***:**

**New methods that incorporate the main and interaction effects of high-dimensional markers and of high-dimensional environmental covariates gave increased prediction accuracy of grain yield in wheat across and within environments**.

**Abstract:**

In most agricultural crops the effects of genes on traits are modulated by environmental conditions, leading to genetic by environmental interaction (G × E). Modern genotyping technologies allow characterizing genomes in great detail and modern information systems can generate large volumes of environmental data. In principle, G × E can be accounted for using interactions between markers and environmental covariates (ECs). However, when genotypic and environmental information is high dimensional, modeling all possible interactions explicitly becomes infeasible. In this article we show how to model interactions between high-dimensional sets of markers and ECs using covariance functions. The model presented here consists of (random) reaction norm where the genetic and environmental gradients are described as linear functions of markers and of ECs, respectively. We assessed the proposed method using data from Arvalis, consisting of 139 wheat lines genotyped with 2,395 SNPs and evaluated for grain yield over 8 years and various locations within northern France. A total of 68 ECs, defined based on five phases of the phenology of the crop, were used in the analysis. Interaction terms accounted for a sizable proportion (16 %) of the within-environment yield variance, and the prediction accuracy of models including interaction terms was substantially higher (17–34 %) than that of models based on main effects only. Breeding for target environmental conditions has become a central priority of most breeding programs. Methods, like the one presented here, that can capitalize upon the wealth of genomic and environmental information available, will become increasingly important.

## Introduction

In the analysis of agricultural data and plant breeding experiments, the development of methods for modeling the interaction between genotypes and environments (G × E) precedes the development of analysis of variance. For instance, Fisher and Mackenzie (1923) suggested modeling the differential responses of genotypes (G) to environments (E) using a multiplicative (product) operator rather than additive models. Yates and Cochran (1938) proposed using a multiplicative operator consisting of a simple regression of a line’s performance on the environmental mean (joint-regression analysis). Years later, other multiplicative operators based on singular value decomposition were proposed and used by Gollob (1968), Mandel (1969), Gauch (1988), Cornelius et al. (1996) and Crossa and Cornelius (1997). Later, Piepho (1998) and Smith et al. (2001, 2005) used this multiplicative operator for modeling G × E but in the context of linear mixed-effect models, and Crossa et al. (2004, 2006) and Burgueño et al. (2008, 2011) considered the use of structured covariance matrices to model G × E in the context of pedigree-based mixed models.

The main effects of genes and of environmental conditions could be modeled by regressing phenotypes on genetic markers and on environmental covariates (ECs; e.g., temperature, soil moisture, solar radiation) concurrently; and G × E can in principle be modeled using interactions between genetic markers and ECs. An example of such an approach is the factorial regression (FR) model (Denis 1988; van Eeuwijk et al. 1996; Vargas et al. 1999, 2001). Models for QTL × environment interaction (Q × E) have been applied both in the context of fixed-effects regression, such as the FR, and using Partial Least Squares (Crossa et al. 1999; Vargas et al. 2006). More recently, these methods were used in a mixed model context (Boer et al. 2007; Malosetti et al. 2004) and later extended to multi-environment multi-trait model settings (Malosetti et al. 2008; Alimi et al. 2013).

With the development of modern genotyping and sequencing technologies, molecular marker information has become high dimensional, with the number of markers (*p*) potentially exceeding by large the number of phenotypic records (*n*) available for model fitting. Similarly, as climatic and agronomic information systems develop, environmental information also becomes high dimensional. The Q × E models discussed above cannot cope with the high-dimensional nature of genomic and EC information. A simple approach would be to introduce a first step where a few ‘significant’ ECs and genomic regions are selected, with only a small subset of markers and ECs used in the final model. However, with variable selection procedures important volumes of information can be lost in the process of selecting markers (Bernardo 2010; Crossa 2012) or ECs, with the undesirable result that large proportions of genetic or environmental signals may be unaccounted for.

To circumvent the limitations of QTL-based models, Meuwissen et al. (2001) proposed using whole genome regression (WGR) methods (also known as models for genomic selection, GS) where information about potentially hundreds of thousands of markers is jointly considered. Such models allow capturing not only major-effect genes but also the contribution of genomic regions with small effects. Implementing these large-*p* with small-*n* regressions is possible using modern shrinkage estimation procedures, and empirical evidence obtained with plant breeding data shows that GS can outperform the predictive power of pedigree-based methods by a sizable amount (de los Campos et al. 2009; Crossa et al. 2010, 2011; Heslot et al. 2012; Pérez et al. 2010). Recently, GS models were extended to multi-trait multi-environment settings. For instance, Burgueño et al. (2012) used a multi-environment version of the genomic best linear unbiased predictor (G-BLUP) where G × E was modeled using genetic correlations, and found that the multi-environment G-BLUP had a much higher prediction accuracy than the single-trait G-BLUP. However, the study of Burgueño et al. (2012) did not incorporate environmental variables to model G × E.

The principles used in GS to model the effects of genetic markers could also be exploited for modeling the main effects of large numbers of ECs, and, in theory, one could also include in the model explicitly all possible contrasts between markers and ECs. However, such an approach would be extremely demanding because the number of contrasts to be considered (and consequently the number of effects to be estimated) grows proportionally to the product of the number of markers and the number of ECs, leading to important statistical and computational challenges.

In this article, we propose a class of random effects models where the main and interaction effects of markers and ECs are introduced using covariance structures that are functions of marker genotypes and ECs. The proposed approach represents an extension of the G-BLUP and can be interpreted as reaction norm model (e.g., Woltereck 1909; Gregorius and Namkoong 1986; Falconer and Mackay 1996; Calus et al. 2002; Calus and Veerkamp 2003; Su et al. 2006) where genetic and environmental gradients are described using a linear regression on genetic markers and on ECs. We evaluated the proposed methods using a data set from Arvalis, consisting of 139 wheat lines evaluated for grain yield in 340 year × location combinations. Genetic and ECs information consisted of 2,395 SNPs and 68 ECs. The prediction accuracy of the proposed models was assessed for two prediction problems: one (CV1) in which models are used to predict the performance of lines that have never been evaluated in field trials (newly developed lines) and a second design (CV2) in which all lines have at least one field evaluation available and the prediction problem was that of predicting performance across environments (i.e., in an incomplete field trial). Predictive correlations were in line with previous reports on grain yield but varied considerably depending on the prediction problem (CV1 or CV2) and the model used. Relative to a model that accounted only for the main effects of markers, environments and ECs, the introduction of interactions between these terms increased the predictive correlation by roughly 35 %, from 0.175 to 0.236 in CV1 and 17 % from 0.439 to 0.514 in CV2. Therefore, we concluded that sizable gains in prediction accuracy can be attained by combining molecular marker information with EC data.

## Materials and methods

### Experimental data set

Data were provided by Arvalis and consisted of a total of 7,876 field records of grain yield collected on 139 commercial lines tested in eight different years (from 2003–10) and 134 locations within northern France, yielding a total of 340 location × year combinations. No further information about the experimental design was available. All trials received fungicide and seed treatments, all locations had a meteorological station within a distance of less than 10 km, and soil characteristics at each location were analyzed. The lines were screened for grain yield (15 % moisture content) and yield components in plots harvested at maturity. Care was taken for the data to have connections across locations and years, with 55, 20, 11, 3, 2 lines being evaluated in at least 50, 100, 150, 200 and 330 location × year combinations.

Lines were genotyped for 3,548 SNPs using an insertion site-based polymorphism technique (Paux et al. 2010). After removing SNPs with minor allele frequency smaller than 3 % and SNPs with more than 10 % of missing values, a total of 2,395 SNPs were still available for analysis. The remaining missing genotypes were imputed using $2\theta_{m}$ where $\theta_{m}$ is the estimated frequency of the allele coded as one at the *m*th marker. A total of 130 ECs that described environmental conditions were collected. These environmental conditions were related to abiotic factors such as temperature, soil type, humidity, radiation and precipitation. Environmental covariates were calculated based on climatic records and soil characteristics, allowing the estimation of water balance.

The phenology of the crop was divided into five phases (ear 1 cm, ear 1 cm to spiking, spiking to flowering, flowering to milk stage and milk stage to harvesting), and environmental descriptors linked to water deficit, the effects of minimum and maximum temperatures, evapotranspiration rate and radiation were defined for each of these phases yielding a total of 130 distinct ECs. Covariates that had more than 30 % of repeated values or more than 0.2 % of values outside the range defined by the mean ± 4 SD were removed. After applying this quality control, a total of 68 ECs were used in the analysis. Both markers and ECs were centered by subtracting the mean of each marker or EC, and standardized to a unit variance by dividing the centered values by the standard deviation of the marker or EC.

### Statistical methods

We begin by describing a set of models that define the building blocks that are later combined to arrive at the sequence of models used for data analysis.

#### Baseline model

The starting point is a model where phenotypes ($y_{ijk}$) are described as the sum of an overall mean ($\mu$) plus random deviations due to the environment ($E_{i}$; *i* = *1*,…,*I*), defined hereinafter as the location × year combination, and the line ($L_{j}$; *j* = *1*,…,*J*), plus an error term ($\varepsilon_{ijk}$; *k* = *1*,…$r_{ij}$). For the random components, we adopted the standard assumption of mixed effects models; therefore:

1 $$y_{ijk} = \mu + E{}_{i} + L_{j} + \varepsilon_{ijk}$$

where $E_{i} \mathop \sim \limits^{\text{IID}} {\text{N}}\left( {0,\sigma_{E}^{2} } \right)$, $L_{j} \mathop \sim \limits^{\text{IID}} {\text{N}}\left( {0,\sigma_{L}^{2} } \right)$ and $\varepsilon_{ijk} \mathop \sim \limits^{\text{IID}} {\text{N}}\left( {0,\sigma_{\varepsilon }^{2} } \right)$,and N(.,.) denotes a normal density and IID stands for independent and identically distributed. In the baseline model, the effects of the different levels of each random effect are independent; therefore, in the model described above there is no borrowing of information across lines or across environments.

#### Introducing genetic markers in the baseline model using G-BLUP

When markers are available, one can consider replacing in (1) the random effect of the line with a regression on marker covariates of the form: $g_{j} = \sum\nolimits_{m = 1}^{p} {x_{jm} b_{m} }$, where $g_{j}$ represents an approximation of the true genetic value of the *j*th line, $x_{jm}$ is the genotype of the *j*th line at the *m*th marker, and $b_{m}$ is the effect of the *m*th marker. Following the standard assumptions of the Ridge-Regression-BLUP model (Habier et al. 2007; VanRaden 2008), we regarded marker effects as IID draws from normal distributions of the form $b_{m} \mathop \sim \limits^{\text{IID}} {\text{N}}\left( {0,\sigma_{b}^{2} } \right)$, *m* = *1*,…,*p*.

From properties of the multivariate normal density we have that the vector ${\mathbf{g}} = {\mathbf{Xb}}$ containing the genomic values of all the lines follows a multivariate normal density with null mean and covariance-matrix ${\text{Cov}}\left( {\mathbf{g}} \right) = {\mathbf{G}}\sigma_{g}^{2},$ where **G** is a genomic relationship matrix whose entries are given by $G_{{jj^{\prime}}} = p^{-1}{\sum\nolimits_{m = 1}^{p} \frac{{\left( {x_{jm} - 2\theta_{m} } \right)\left( {x_{{j^{\prime}m}} - 2\theta_{m} } \right)} }{2\theta_m(1-\theta_m)}}$. Here, $\theta_{m}$ is the estimated frequency of the allele whose number of copies at the *j*th individual is counted in $x_{jm}$. Centering (i.e., subtracting $2\theta_{m}$ from the genotype codes) or standardization (i.e., dividing each marker covariate by $\sqrt{2\theta_{m} \left( {1 - \theta_{m} } \right)}$) are not strictly needed; however standardization allows interpreting $\sigma_{g}^{2}$ as a genomic variance. The matrix **G** is a marker-derived genomic relationship matrix, and its entries converge (as the number of independently segregating loci increases) to twice the kinship coefficient between lines. Collecting the above-mentioned assumptions, we have the standard G-BLUP model plus a random environmental effect (E) yielding the EG model:

2 $$y_{ijk} = \mu + E{}_{i} + g_{j} + \varepsilon_{ijk}$$

with $$E_{i} \mathop \sim \limits^{\text{IID}} {\text{N}}\left( {0,\sigma_{E}^{2} } \right)$$, $${\mathbf{g}}\sim {\text{N}}\left( {{\mathbf{0,G}}\sigma_{g}^{2} } \right)$$ and $$\varepsilon_{ijk} \mathop \sim \limits^{\text{IID}} {\text{N}}\left( {0,\sigma_{\varepsilon }^{2} } \right)$$.

Note that unlike (1), the effects of the level of the random effects ${\mathbf{g}} = \left( {g_{1} , \ldots ,g_{J} } \right)^{\prime }$ are now correlated according to the off-diagonal values of **G**; therefore, in the model in expression (2), there is potentially borrowing of information across lines. This allows, for example, predicting the performance of lines that have not been evaluated in any field trial.

#### Extending G-BLUP with addition of environmental covariates

Using the same principles used in G-BLUP, we can now replace in (2) the environmental effects ($E{}_{i}$) with a random regression on the ECs (**W**) that describes the environmental conditions faced by each line in each environment, that is: $w_{ij} = \sum\nolimits_{q = 1}^{Q} {W_{ijq} \gamma_{q} }$, where $W_{ijq}$ is the value of the *q*th EC evaluated in the *ij*th environment × line combination, $\gamma_{q}$is the main effect of the corresponding EC, and *Q* is the total number of ECs. As before, we regarded the effects of the ECs as IID draws from normal densities, that is, $\gamma_{q} \mathop \sim \limits^{\text{IID}} {\text{N}}\left( {0,\sigma_{\gamma }^{2} } \right)$. Consequently, the vector ${\mathbf{w}} = {\mathbf{W\gamma }}$ follows a multivariate normal density with null mean and a covariance matrix proportional to ${\varvec{\Omega}}$ whose entries are computed as those of the G-matrix but using ECs instead of markers. This covariance structure describes the similarity between environmental conditions in a similar way that **G** describes genetic similarity between lines. Therefore, when the effects of the environments in (2) are replaced with ${\mathbf{w}} = {\mathbf{W\gamma }}$, the model becomes

3 $$y_{ijk} = \mu + w{}_{ij} + g_{j} + \varepsilon_{ijk}$$

where $${\mathbf{w}}\sim {\text{N}}\left( {{\mathbf{0}},{\varvec{\Omega}}\sigma_{w}^{2} } \right)$$, $${\mathbf{g}}\mathop \sim \limits^{{}} N\left( {{\mathbf{0,G}}\sigma_{g}^{2} } \right)$$ and $$\varepsilon_{ijk} \mathop \sim \limits^{\text{IID}} N\left( {0,\sigma_{\varepsilon }^{2} } \right)$$.

In the model of expression (3) the covariance matrices **Ω** and **G** permit the borrowing of information between environments and between lines, respectively.

The ECs may not fully describe differences across environments, perhaps because some relevant ECs were not measured or because of model miss-specification (e.g., non-linear effects of ECs on the trait of interest). Similarly, because of imperfect linkage disequilibrium (LD) between markers and genes at causal loci or because of model miss-specification (e.g., interactions between alleles that are unaccounted for), the regression on markers may not fully describe genetic differences among lines. One possibility is to combine models (1) and (3) into a single model of the following form:

4 $$y_{ijk} = \mu + E_{i} + w{}_{ij} + L_{j} + g_{j} + \varepsilon_{ijk}$$

where $E_{i} \mathop \sim \limits^{\text{IID}} N\left( {0,\sigma_{E}^{2} } \right)$, ${\mathbf{w}}\mathop \sim \limits^{{}} N\left( {{\mathbf{0}},{\varvec{\Omega}}\sigma_{w}^{2} } \right)$, $L_{j} \mathop \sim \limits^{IID} N\left( {0,\sigma_{L}^{2} } \right)$, ${\mathbf{g}}\mathop \sim \limits^{{}} N\left( {{\mathbf{0,G}}\sigma_{g}^{2} } \right)$ and $\varepsilon_{ijk}^{{}} \mathop \sim \limits^{IID} N\left( {0,\sigma_{\varepsilon }^{2} } \right)$. In the model in Eq. (4), both environmental and line effects are partitioned into two components, one that is explained by regression on covariates (either markers, $g_{j}$, or ECs, $w{}_{ij}$) and deviations that represent variation attributable to lines or environments and cannot be explained by regression on markers ($L_{j}$) or ECs ($E_{i}$).

##### Incorporating interactions between markers and environmental covariates

So far we have presented models that account for the main effects of markers (G) and the main effects of ECs (W) without accounting for possible interactions between markers and ECs. In principle, first order interactions between markers and ECs can be incorporated by first constructing all possible contrasts (for *p* markers and *Q* ECs there will be *p* × *Q* contrasts) and then including these contrasts as predictors in the model. However, when this approach is used, the number of effects to be estimated can be extremely large. For instance, in our case a total of 162,860 terms are required to model first order interactions between 68 ECs and 2,395 SNPs. Modeling interactions in such an explicit way becomes unfeasible when either *p* or *Q* are large. To circumvent this problem, we propose incorporating interactions using covariance structures.

The covariance function generated by the interaction terms will depend on the mode of interaction (which markers interact with which covariates, degree of interactions, etc.). Here we consider the case of a first order multiplicative model where the interaction is the product of two random linear scores: a genetic score, $\tilde{g}_{j} = \sum\nolimits_{m = 1}^{p} {x_{jm} \tilde{b}_{m} }$, and an environmental score, $\tilde{w}_{ij} = \sum\nolimits_{q = 1}^{Q} {W_{ijq} \tilde{\gamma }_{q} }$. To be consistent with our previous assumptions, we assume that both the $\tilde{b}_{m}$’s and the $\tilde{\gamma }_{q}$ are IID, zero-mean, random variables. We further assume that the $\tilde{b}_{m}$’s and the $\tilde{\gamma }_{q}$’s are independent. With these assumptions we have: $E\left[ {\tilde{g}_{j} } \right] = E\left[ {\tilde{w}_{ij} } \right] = 0$, ${\text{Cov}}\left( {\tilde{g}_{j} ,\tilde{g}_{{j^{\prime}}} } \right) \propto G_{{jj^{\prime}}}$ ${\text{Cov}}\left( {\tilde{w}_{ij} ,\tilde{w}_{{i^{\prime}j^{\prime}}} } \right) \propto \varOmega_{{ij,i^{\prime}j^{\prime}}}$ and ${\text{Cov}}\left( {\tilde{g}_{j} ,\tilde{w}_{ij} } \right) = 0$ where $G_{{jj^{\prime}}}$and $\varOmega_{{ij,i^{\prime}j^{\prime}}}$ are entries of **G** and of ${\varvec{\Omega}}$, respectively.

Now consider the random process obtained by multiplying both random scores: $gw_{ij} = \tilde{g}_{j} \times \tilde{w}_{ij}$. The expected value of this process is $E\left[ {gw_{ij} } \right] = E\left[ {\tilde{g}_{j} \times \tilde{w}_{ij} } \right] = E\left[ {\tilde{g}_{j} } \right] \times E\left[ {\tilde{w}_{ij} } \right] = 0$ and the covariance function is:

$$\begin{gathered} {\text{Cov}}\left[ {gw_{ij} ,gw_{{i^{\prime}j^{\prime}}} } \right] = E\left[ {gw_{ij} \times gw_{{i^{\prime}j^{\prime}}} } \right] - E\left[ {gw_{ij} } \right]E\left[ {gw_{{i^{\prime}j^{\prime}}} } \right] \hfill \\ \, = E\left[ {gw_{ij} \times gw_{{i^{\prime}j^{\prime}}} } \right] \hfill \\ \, = E\left[ { \tilde{g}_{j} \times \tilde{w}_{ij} \times \tilde{g}_{{j^{\prime}}} \times \tilde{w}_{{i^{\prime}j^{\prime}}} } \right] \hfill \\ \, = E\left[ {\tilde{g}_{j} \times \tilde{g}_{{j^{\prime}}} } \right]E\left[ {\tilde{w}_{ij} \times \tilde{w}_{{i^{\prime}j^{\prime}}} } \right] \hfill \\ \, = {\text{Cov}}\left[ {\tilde{g}_{j} ,\tilde{g}_{{j^{\prime}}} } \right]Cov\left[ {\tilde{w}_{ij} ,\tilde{w}_{{i^{\prime}j^{\prime}}} } \right] \hfill \\ \, \propto G_{{jj^{\prime}}} \varOmega_{{iji^{\prime}j^{\prime}}} \hfill \\ \end{gathered}$$

Therefore, the covariance function of $gw_{ij} = \tilde{g}_{j} \times \tilde{w}_{ij}$ is simply the cell by cell product (known as the Hadamard or Schur product) of $G_{{jj^{\prime}}}$ and $\varOmega_{{iji^{\prime}j^{\prime}}}$, the entries of **G** and ${\varvec{\Omega}}.$ The terms $G_{{jj^{\prime}}}$ measure the degree of genetic similarity between lines, and $\varOmega_{{iji^{\prime}j^{\prime}}}$ measures the degree of similarity among environmental conditions. Whenever $\varOmega_{{iji^{\prime}j^{\prime}}}$ or $G_{{jj^{\prime}}}$ are close to zero, the product of the two will be close to zero; therefore, resemblance between records due to interaction term requires resemblance both at the genetic and EC level. These types of covariance functions are not new to quantitative genetic methods; indeed, Cockerham (1954) and Kempthorne (1954) arrived at these type of covariance functions when studying the degree of resemblance between relatives generated by epistatic interactions (e.g., additive by additive and additive by dominance). Also, as stated, the multiplicative approach above-described can be viewed as a reaction norm where the genetic ($\tilde{g}_{j}$) and environmental ($\tilde{w}_{ij}$) values, or gradients, are replaced with regressions on markers and on ECs, respectively.

When data involve multiple phenotypic records per line, the genetic covariance structure of additive effects is ${\mathbf{Z}}_{g} {\mathbf{GZ^{\prime}}}_{g}$, where ${\mathbf{Z}}_{g}$ is an incidence matrix for the vector of additive genetic effects. In this case, the covariance structure of the vector of interaction terms ${\mathbf{gw}} = \left\{ {gw_{ij} } \right\}$ is the Hadamard product of ${\mathbf{Z}}_{g} {\mathbf{GZ^{\prime}}}_{g}$ and ${\varvec{\Omega}}$, denoted here as $\left[ {{\mathbf{Z}}_{g} {\mathbf{GZ^{\prime}}}_{g} } \right] \circ {\varvec{\Omega}}$.

Using the results presented above, we then extended the models above-described by adding a term representing interactions between markers and ECs. For instance, we can extend the model in expression (3) as follows:

5 $$y_{ijk} = \mu + w{}_{ij} + g_{j} + gw_{ij} + \varepsilon_{ijk}$$

with $${\mathbf{w}}\mathop \sim \limits^{{}} N\left( {{\mathbf{0}},{\varvec{\Omega}}\sigma_{w}^{2} } \right)$$, $${\mathbf{g}}\mathop \sim \limits^{{}} N\left( {{\mathbf{0,G}}\sigma_{g}^{2} } \right)$$, $${\mathbf{gw}}\sim N\left( {{\mathbf{0,}}\left[ {{\mathbf{Z}}_{g} {\mathbf{GZ^{\prime}}}_{g} } \right] \circ {\varvec{\Omega}}\sigma_{gw}^{2} } \right)$$, $$\varepsilon_{ijk} \mathop \sim \limits^{\text{IID}} N\left( {0,\sigma_{\varepsilon }^{2} } \right)$$.

#### Incorporating interactions between markers and environments

Because of imperfect LD between alleles at markers and alleles at causal loci, markers may not fully account for genetic differences between lines. Similarly, ECs may not fully account for differences due to environmental conditions. Therefore, some proportion of the G × E may not be fully captured by the interaction term $gw_{ij}$. To account for this, one possibility is to expand any of the previously presented models by including an interaction term between environments (*E*_*i*_) and the random effect of the markers (*g*_*j*_). This model is obtained by including the interaction term $gE_{ij}$ in either (2) or (5). Following a procedure similar to that used to obtain (5), we have that the covariance structure generated by $gE_{ij}$ is proportional to the Hadamard product $\left[ {{\mathbf{Z}}_{g} {\mathbf{GZ^{\prime}}}_{g} } \right] \circ \left[ {{\mathbf{Z}}_{E} {\mathbf{Z^{\prime}}}_{E} } \right]$, where ${\mathbf{Z}}_{E}$ represents the incidence matrix for the effects of environments (i.e., the matrix that connects the phenotypes with environments). For instance, adding to the model described in (2), we have a model that accounts for main effects of markers, main effects of environments and the interactions between markers and environments:

6 $$y_{ijk} = \mu + E{}_{i} + g_{j} + gE_{ij} + \varepsilon_{ijk}$$

with $$E_{i} \mathop \sim \limits^{\text{IID}} N\left( {0,\sigma_{E}^{2} } \right)$$, $${\mathbf{g}}\sim N\left( {{\mathbf{0,G}}\sigma_{g}^{2} } \right)$$, $${\mathbf{gE}}\sim N\left( {{\mathbf{0,}}\left[ {{\mathbf{Z}}_{g} {\mathbf{GZ^{\prime}}}_{g} } \right] \circ \left[ {{\mathbf{Z}}_{E} {\mathbf{Z^{\prime}}}_{E} } \right]\sigma_{gE}^{2} } \right)$$, $$\varepsilon_{ijk} \mathop \sim \limits^{\text{IID}} N\left( {0,\sigma_{\varepsilon }^{2} } \right)$$.

##### Phenotypic and genetic correlations

The models above-described, and others that could be constructed by combining the random effects of each of the models listed before, impose specific forms on the phenotypic and genetic correlation functions. In these models, correlation depends on the variance parameters of the model as well as on the degree of environmental and genetic similarity. To illustrate, we present the derivation of the phenotypic and genetic correlations for the model defined by Eq. (5), similar steps can be followed to derive the covariance and correlation functions implied by any of the above-described models. For the model defined by Eq. (5) the covariance between phenotypic records of line *j* measured under environmental conditions *ik* and *i*′*k*′ is,

$$\begin{gathered} {\text{Cov}}\left( {y_{ijk} ,y_{{i^{\prime}jk^{\prime}}} } \right) = {\text{Cov}}(\mu + w{}_{ij} + g_{j} + gw_{ij} + \varepsilon_{ijk} ,\mu + w{}_{{i^{\prime}j}} + g_{j} + gw_{{i^{\prime}j}} + \varepsilon_{{i^{\prime}jk^{\prime}}} ) \hfill \\ \, = \varOmega_{{ij,i^{\prime}j}} \sigma_{w}^{2} + {\text{G}}_{jj} \sigma_{g}^{2} + \varOmega_{{ij,i^{\prime}j}} {\text{G}}_{jj} \sigma_{gw}^{2}, \hfill \\ \end{gathered}$$

and the phenotypic and genetic correlation functions are then given by:

$$\rho_{y(ijk,i'jk')} = \frac{{\varOmega_{{ij,i^{\prime}j}} \sigma_{w}^{2} + {\text{G}}_{jj} \sigma_{g}^{2} + \varOmega_{{ij,i^{\prime}j}} {\text{G}}_{jj} \sigma_{gw}^{2} }}{{\sqrt {\varOmega_{ij,ij} \sigma_{w}^{2} + {\text{G}}_{jj} \sigma_{g}^{2} + \varOmega_{ij,ij} {\text{G}}_{jj} \sigma_{gw}^{2} + \sigma_{\varepsilon }^{2} } \sqrt {\varOmega_{{i^{\prime}j,i^{\prime}j}} \sigma_{W}^{2} + {\text{G}}_{jj} \sigma_{g}^{2} + \varOmega_{{i^{\prime}j,i^{\prime}j}} {\text{G}}_{jj} \sigma_{gw}^{2} + \sigma_{\varepsilon }^{2} } }}$$

and$$\rho_{g(ijk,i'jk')} = \frac{{{\text{G}}_{jj} \sigma_{g}^{2} + \varOmega_{{ij,i^{\prime}j}} {\text{G}}_{jj} \sigma_{gw}^{2} }}{{\sqrt {{\text{G}}_{jj} \sigma_{g}^{2} + \varOmega_{ij,ij} {\text{G}}_{jj} \sigma_{gw}^{2} } \sqrt {{\text{G}}_{jj} \sigma_{g}^{2} + \varOmega_{{i^{\prime}j,i^{\prime}j}} {\text{G}}_{jj} \sigma_{gw}^{2} } }}$$, respectively.

### Data analysis

#### Models

Using the random effects included in models (1)–(6) as building blocks, we defined a sequence of models that were used for empirical data analysis. The effects included in each of the seven models in our sequence are described in Table 1. The columns in Table 1 give the random effects considered, for main effects (E, L, G and W) and for interactions (G × E and G × W).

**Table 1:** Main effect and interaction of the seven models used to fit the data set

Model abbreviation	Factors included					
	Main effect				Interaction	
	E	L	G	W	G × E	G × W
EL	X	X				
EG	X		X			
ELW	X	X		X		
EGW	X		X	X		
EGW-G × E	X		X	X	X	
EGW-G × W	X		X	X		X
EGW-G × WG × E	X		X	X	X	X

*E* environment, *L* line, *G* marker covariates, *W* environmental covariates, *G* *×* *E* interaction between environments and markers, *G* *×* *W* interactions between markers and ECs

Each of the models described in Table 1 were fitted to the full data set using the computational methods described in de los Campos et al. (2010) which were recently implemented in the R-package BGLR (de los Campos and Perez-Rodriguez 2013). All the statistical analyses were done using the R-software R Core Team (2013).

#### Assessment of prediction accuracy

Following Burgueño et al. (2012), we considered two distinct predictions problems: in the first one (hereinafter denoted as CV1), we measured the ability of the model to predict the performance of lines that have not yet been evaluated in any field trial (newly released varieties). In the second design (CV2), we assessed the ability of models to predict the performance of lines using data collected in other environments. This design mimics the prediction problem encountered in incomplete field trials and was also used by Burgueño et al. (2012). In CV1 we randomly assigned lines to folds; this assures that all the records of a given line are assigned to the same fold. On the other hand, in CV2 we randomly assigned individual plot records to folds; with this setting individual records of a given line are potentially assigned to different folds. Table 2 gives a graphical representation of the two prediction problems where, for example, CV1 aims to predict the performance of Line 3, (unobserved in all environments) in environments E1–E5, using phenotypic records from Lines 1, 2, 4 and 5 (observed in all environments). On the other hand, in CV2, the aim is to predict the performance of Lines 1, 2, 3, 4 and 5 in environments E2, E3, E5, E4 and E1, respectively. Naturally, CV1 presents a much more difficult prediction problem because it is not possible to borrow information within lines (Line 3, in our case) from any other environment. We implemented CV1 and CV2 in a tenfold design.

**Table 2:** Two hypothetical cross-validation schemes (CV1 and CV2) for five lines (Lines 1–5) and five environments (E1–E5)

CV1						CV2				
	E1	E2	E3	E4	E5	E1	E2	E3	E4	E5
Line 1	*Y*_11_	*Y*_12_	*Y*_13_	*Y*_14_	*Y*_15_	*Y*_11_	NA	*Y*_13_	*Y*_14_	*Y*_15_
Line 2	*Y*_21_	*Y*_22_	*Y*_23_	*Y*_24_	*Y*_25_	*Y*_21_	*Y*_22_	NA	*Y*_24_	*Y*_25_
Line 3	NA	NA	NA	NA	NA	*Y*_31_	*Y*_32_	*Y*_33_	*Y*_34_	NA
Line 4	*Y*_41_	*Y*_42_	*Y*_43_	*Y*_44_	*Y*_45_	*Y*_41_	*Y*_42_	*Y*_43_	NA	*Y*_45_
Line 5	*Y*_51_	*Y*_52_	*Y*_53_	*Y*_54_	*Y*_55_	NA	*Y*_52_	*Y*_53_	*Y*_54_	*Y*_55_

Lines with unobserved phenotypic data in the cross-validation scheme are indicated by NA (not available); lines with observed values in environments are denoted as *Y*_*ji*_ for (*i*, *j* = 1, 2, 3, 4, 5)

#### Target of prediction

We focus on assessing the ability of the model to predict performance of lines within environments; therefore, prediction accuracy was assessed based on the ability of each model to predict phenotypic yields, after accounting for the main effects of the environment. To this end, both the observed phenotypic records and the CV-derived predicted performance were pre-corrected with (CV-derived) estimates of the main effects of the environments and of the ECs, that is, $\hat{\eta }_{ij} = \hat{E}_{j} + \hat{w}_{ij}$, derived from the most comprehensive model (EGW-G × WG × E; see Table 1) and in the corresponding fold of the CV. The adjusted phenotype ($\tilde{y}_{ijk} = y_{ijk} - \hat{\eta }_{ij}$) was then compared with the corrected CV-predictions derived from each of the models listed in Table 1 ($\tilde{\hat{y}}_{ijk} = \hat{y}_{ijk} - \hat{\eta }_{ij}$), where $\hat{y}_{ijk}$ is a CV-derived predicted yield. The correlation between adjusted phenotypes and adjusted predictions was computed using Pearson’s product-moment correlation coefficient evaluated on the entire vector of adjusted predictions and adjusted phenotypes. As measures of uncertainty about estimates of correlations we present: (a) confidence intervals (CIs) based on a large-sample formula for the SE of the correlation coefficient, $\hat{\rho } \pm 1.96\sqrt {\frac{{1 - \hat{\rho }^{2} }}{n - 2}}$ where $\hat{\rho }$ is the estimated correlation coefficient, and (b) CIs derived using a bootstrap procedure implemented by re-sampling the entries of the vectors of adjusted phenotypes $\left({\tilde{\mathbf{y}}} = \left\{ {y_{ijk} - \hat{\eta }_{ij} } \right\}\right)$ and of adjusted predictions $\left({\tilde{\hat{\mathbf{y}}}} = \left\{ {\tilde{\hat{y}}_{ijk} } \right\}\right)$ 10,000 times. Finally, we present a rank-based measure of association obtained by categorizing ${\tilde{\mathbf{y}}}$ and ${\tilde{\hat{\mathbf{y}}}}$ into classes based on percentiles of the empirical distribution of each of these variables and report the conditional distribution of the categories defined by ${\tilde{\mathbf{y}}}$ given the categories defined by ${\tilde{\hat{\mathbf{y}}}}$.

## Results

**Fig. 1:**
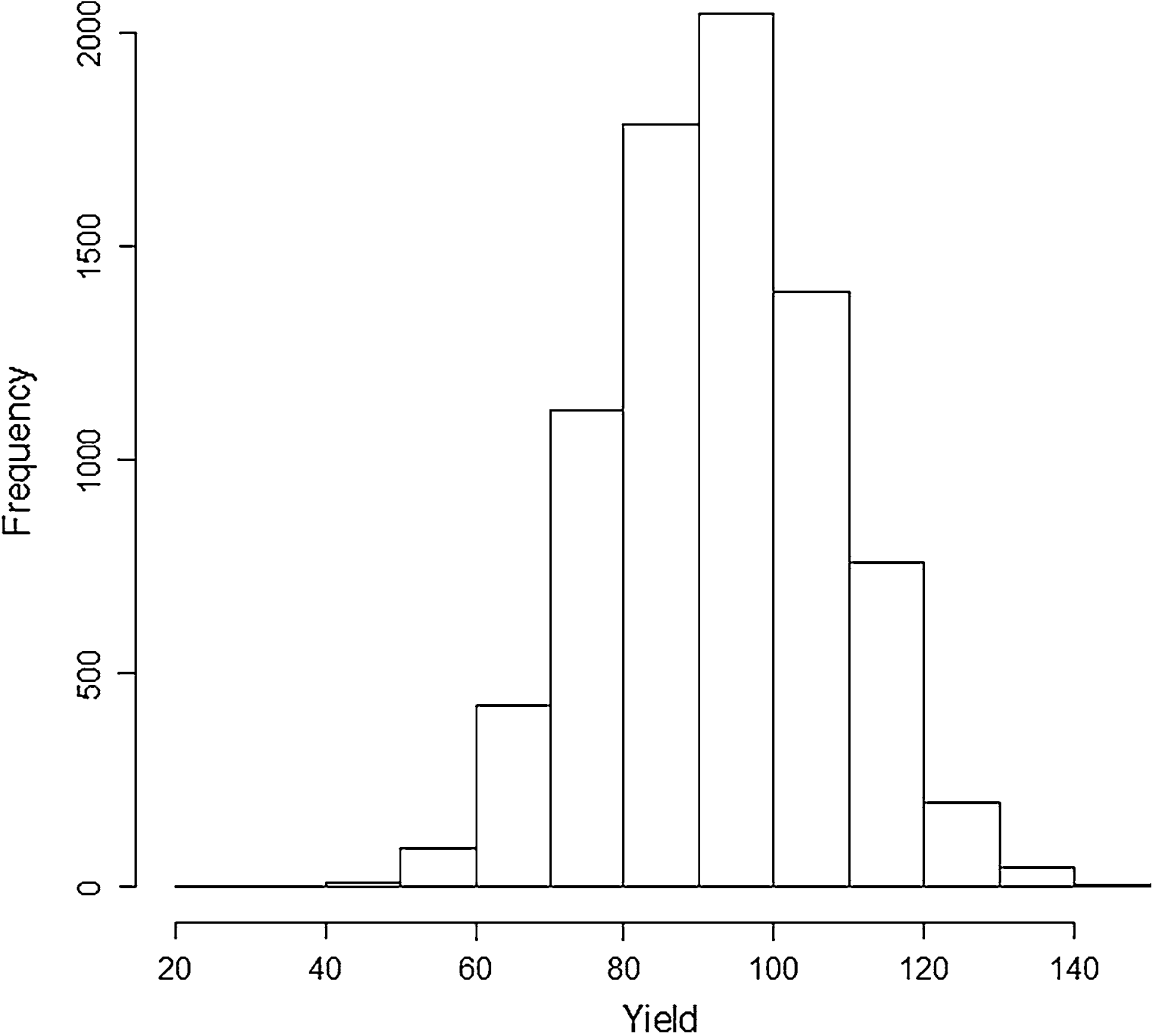
Histogram of grain yield in quintals per hectare

Figure 1 gives a histogram of grain yield. The empirical yield distribution was close to normal, with an average of 92.5 quintals per hectare and a standard deviation of 15, corresponding a coefficient of variation of 16 %. The distribution of the minor allele frequencies was close to uniform in the (0.03–0.5) range.

**Fig. 2:**
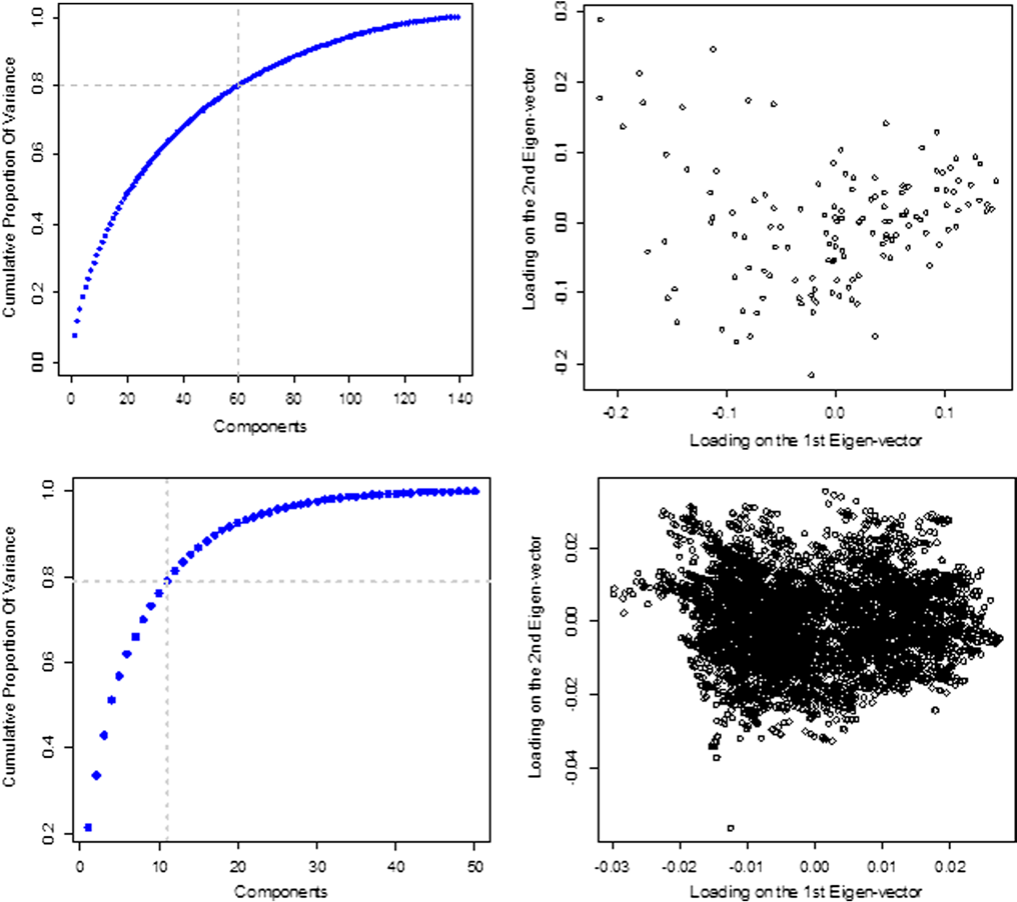
Scree plot (*left panel*) and loadings of the first two eigenvectors (*right panel*) of the covariance matrices derived from markers (*top panel*) and from environmental covariates (*lower panel*)

Figure 2 gives the scree plot of the eigenvalues (left panel) and the loadings of the first two eigenvectors (right panel) of the eigenvalue decomposition of the matrices **G** and **Ω** (top and lower panels, respectively). The first two eigenvectors of the marker-derived genomic relationship matrix showed some but not strong evidence of population stratification. The proportion of the variance of marker genotypes explained by the first two eigenvectors was 11.5 %, and the top 60 eigenvectors (of a total 138 eigenvectors with non-zero eigenvalues) were needed to explain 80 % of the variance of genotypes, suggesting that we are in the presence of a relatively diverse set of lines. This was expected because lines in this data set come from different breeding plans. The scree plot of the eigenvalues of **Ω** shows that at least 11 eigenvectors are needed to account for ~80 % of the variance observed in environmental covariates. Two main clusters seem to be separated by the first eigenvector, but the separation is not very clear, suggesting that environmental variation is better characterized by a continuum of variability in environmental conditions rather than by clusters of environments.

### Estimates of variance components

Table 3 gives estimates of variance components derived from the full data analysis. The main effect of environments (E) was the random effect that explained the largest proportion of the yield variance, with estimated posterior means between 144 and 200, depending on the model. However, as noted earlier, from the point of view of predicting ranks within an environment, variability due to the mean of the environment becomes a nuisance; therefore, we also report (in Table 3) the proportional contribution of each random effect to within-environment variance, that is, relative to the total variance minus the variance due to the main effects of environment.

**Table 3:** Estimated variance components

Model	Variance component												
	Estimate							Percentage of the within-environment variance^a^					
	E	L	G	W	G × E	G × W	Res.	L	G	W	G × E	G × W	Res
EL	199.7	12.3					22.6	35.2					64.8
EG	199.0		14.9				22.6		39.7				60.3
EW	153.5			23.7			27.7			46.1			53.9
ELW	143.7	12.9		24.9			22.1	21.5		41.6			36.9
EGW	145.6		14.6	23.3			22.1		24.3	38.8			36.8
EGW-G × E	148.0		14.4	20.2	5.9		16.4		25.3	35.5	10.4		28.8
EGW-G × W	146.7		12.8	22.3		5.3	18.3		21.8	38.0		9.0	31.2
EGW-G × WG × E	148.6		12.7	19.8	3.9	5.0	14.9		22.6	35.2	6.9	8.9	26.5

*E* environment, *L* line, *G* genomic [marker] information, *W* environmental covariate [EC] information, *G* *×* *E* genotype × environment and G × W genotype × EC interaction, and *Res* model residual

^a^Relative to the total variance minus the variance due to main effect of the environment

When information on ECs was incorporated into the model, the estimated variance due to environments (E) diminished from roughly 200 (either model EL or model EG) to values slightly smaller than 150 (e.g., 143.7, in model ELW and 145.6 in model EGW), indicating that the ECs (W) captured a sizable proportion of across environment variation. However, a large proportion of the total variance was attributable to environments (E), even after including all ECs in the model.

The estimated variance due to lines (L; e.g., 12.3 in model EL) and that associated with regression on SNPs (G; e.g., 14.9 in model EG) were very similar, but slightly larger for G, suggesting that markers are able to capture a sizable proportion, if not all, of the variability due to the main effects of genotypes.

The inclusion of interaction terms (G × E, G × W, and both G × W and G × E) induced a reduction in the estimated residual variance of about 33 % (from an estimated residual variance of 22.1 in model EGW to 14.9 in model EGW-G × WG × E) indicating that some components of differences across lines and environmental conditions cannot be fully captured by the main effects of markers, environments and ECs. Estimates from our most comprehensive model (EGW-G × WG × E) suggest that, of the within-environment variability, roughly 23 % can be explained by main effects of markers, 35 % by main effects of ECs, 16 % by interaction terms, and 26 % by factors unaccounted for (residuals). The proportion of within-environment variation that is explained by interactions (16 %) is not negligible and reflects the importance of considering such interactions in models for genome-enabled prediction. However, it is worth noting that the variance due to main effects of markers and of ECs increased when interactions were omitted, suggesting that some proportion of the variance due to interactions may be captured by main effects if interactions are omitted.

### Assessment of prediction accuracy

The estimated correlations between corrected phenotypes and predictions obtained in CV1 and CV2 are shown in Table 4. This table also provides an estimated 95 % Confident Interval (CI) estimated using two different procedures. In CV1 the prediction problem consisted of predicting the performance of lines with no previous records (newly released lines) and the correlation ranged from very small values (0.09 for the model not including markers, EL) to 0.236 (most comprehensive model, EGW-G × WG × E). A null correlation is expected for CV1 in the case of models that do not include markers or environmental covariates because in CV1 predictions are derived without using records of the lines being predicted. In such contexts, borrowing information from other lines in the same or other environments takes place through markers and environmental covariate information. When the main effects of markers and ECs were included (model EGW), the predictive correlation was 0.175. Further, when interactions were included, the correlation rose to 0.236; this is a 35 % increase in correlation achieved by adding interaction terms.

**Table 4:** Estimated correlations between adjusted phenotypes and cross-validation prediction for each of the seven models for cross-validation CV1 (prediction without using phenotypic records of the lines whose performance is predicted, i.e., prediction for un-tested lines) and CV2 (prediction in incomplete field trials)

Models	CV1			CV2		
	Estimate	95 % CI		Estimate	95 % CI	
		Par.^a^	Non-P.^b^		Par.^a^	Non-P.^b^
EL	0.090	[0.068; 0.112]	[0.063; 0.117]	0.425	[0.405; 0.445]	[0.404; 0.447]
EG	0.191	[0.169; 0.213]	[0.167; 0.215]	0.426	[0.406; 0.446]	[0.404; 0.448]
ELW	−0.027	[−0.049; −0.005]	[−0.050; −0.004]	0.438	[0.418; 0.458]	[0.416; 0.459]
EGW	0.175	[0.153; 0.197]	[0.151; 0.198]	0.439	[0.419; 0.459]	[0.417; 0.460]
EGW-G × E	0.209	[0.187; 0.231]	[0.185; 0.232]	0.454	[0.434; 0.474]	[0.432; 0.476]
EGW-G × W	0.214	[0.192; 0.236]	[0.191; 0.237]	0.506	[0.486; 0.525]	[0.495; 0.525]
EGW-G × WG × E	0.236	[0.215; 0.257]	[0.213; 0.259]	0.514	[0.495; 0.533]	[0.494; 0.535]

^a^Computed using $\hat{\rho } \pm 1.96\sqrt {\frac{{1 - \hat{\rho }^{2} }}{n - 2}}$, where $\hat{\rho }$ is the estimated correlation and *n* is the number of records used to compute the correlation

^b^Obtained by Bootstrapping 10,000 times the vectors of CV-adjusted predictions and CV-adjusted phenotypes

The prediction correlations obtained in CV2 were much higher than those observed in CV1; this was to be expected because in CV2 predictions can benefit from records (collected in other environments) of the line whose performance we want to predict. The predictive correlation for the baseline model (EL) was 0.425. When ECs were added to the model, there was a 3–4 % increase in correlation (from about 0.425–6 in either model EL or model EG to 0.438–9 in model ELW and EGW, respectively). But the most notorious increase in correlation occurred when interactions (both G × E and G × W) were added to the model. Our most comprehensive model yielded a predictive correlation of 0.514, which is a 21 % increase in correlation over the baseline model.

**Fig. 3:**
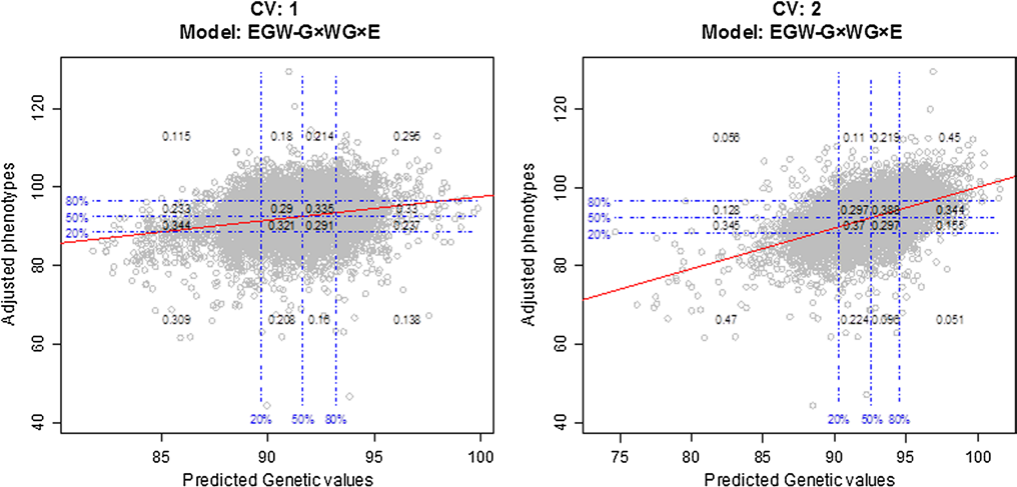
Adjusted phenotype versus adjusted cross-validation predictions, derived from the most comprehensive model (EGW-G × WG × E) in two cross-validation designs (CV 1: prediction without using phenotypic records of the lines whose performance is predicted, i.e., prediction of un-tested lines; and CV 2: prediction in incomplete field trials). *Horizontal* and *vertical dashed lines * give the 20, 50 and 80 % empirical percentiles of the variables in the vertical and horizontal axes, and the *numbers* inside the grid give the observed proportions of each of four groups defined by the percentiles displayed for observed adjusted yield, given the groups defined in the *horizontal line* (predictions)

An alternative way of assessing the ability of a model to predict yet-to-be-observed phenotypes is to evaluate the agreement/disagreement of rankings based on observed and predicted performance. Figure 3 shows, for the most comprehensive model (EGW-G × WG × E), CV-adjusted yields (vertical axis) versus CV-adjusted predictions (horizontal axis) for CV1 (right panel) and CV2 (left panel) designs. In each of the figures, we superimposed a grid defined based on the empirical percentiles of the variables in the horizontal and vertical axes (adjusted predictions and adjusted phenotypes). The numbers within each cell in the grid give the (estimated) conditional probability of the observed ranking (based on the corrected phenotypes, the variable in the vertical axis), given the predicted ranking (based on genomic prediction). For instance, in CV1, if one were to recommend, based on predicted performance, the top 20 % lines for either breeding or agronomic purposes, we estimate that roughly 63 % of these lines would have a performance above the median. For the prediction problem of CV2, 79 % of the lines ranked in the top 20 % (based on predictions) did have observed performance above the median.

## Discussion

Genotype × environment interaction is ubiquitous in agricultural crops. In genomic models G × E can be modeled by including interactions between markers and environments or ECs (e.g., Denis 1988; van Eeuwijk et al. 1996; Vargas et al. 1999, 2001; Malosetti et al. 2004; Boer et al. 2007). However, when the number of markers and ECs is large, modeling explicitly all possible interactions between markers and ECs becomes infeasible because the number of contrasts to be considered increases proportional to the product of the number of markers and of ECs. To circumvent this problem, we propose a variance components approach that allows modeling the main and interaction effects of large numbers of markers and of ECs jointly using covariance structures. In our model, the main effects of markers and ECs are modeled using the same principles used in the standard G-BLUP, and the interaction terms are described using a multiplicative model, equivalent to a reaction norm model where the genetic and environmental gradients are described using regressions on markers and on ECs, respectively.

The multiplicative model used to describe interactions induces a covariance function that is the Hadamard product of two covariance structures: one defining similarity based on markers (the **G** matrix used in standard G-BLUP) and one describing similarity between records due to ECs (**Ω**). This type of covariance structures is not new to quantitative genetics; indeed, it has emerged before in the analysis of infinitesimal models for various forms of interactions between alleles at different loci (e.g., Cockerham 1954; Kempthorne 1954). Importantly, once the covariance functions for main and interaction effects are defined, the implementation of the proposed models is straightforward using Bayesian or other likelihood-based (e.g., REML) methods.

The proposed model was used to analyze data set consisting of 7,876 records of grain yield collected on 139 commercial lines tested in eight different years (from 2003–10) and various locations within northern France. We found that in our data set roughly 16 % of within-environment variation of wheat grain yield was explained by interactions either between markers and ECs (9 %) or between markers and environments (7 %). Moreover, when interaction terms were included in the model, we observed a 17–34 % (depending on the validation design) increase in prediction correlation in cross-validation. This suggests that introducing interactions between markers and environmental conditions can increase the proportion of variance accounted for by the model and, more importantly, it can increase prediction accuracy. The increase in prediction accuracy with the inclusion of environmental information represents a very promising result and has important implications both for breeding as well as for agronomic recommendations.

Burgueño et al. (2012) compared the prediction accuracy for wheat GY (defined as average of two replicates) of models, with and without G × E. In a CV similar to our CV2, Burgueño et al. (2012) reported prediction correlations of 0.475 (for a model without G × E) to 0.556 (for a model with G × E). These values are similar to the values we obtained in CV2 for models with (0.439 for model EGW) and without G × E (0.514 for model EGW-G × E-G × W). Our prediction correlations were only slightly smaller and this is expected because the trait analyzed in Burgueño et al. (2012) was the average yield of two replicates, while in our case we analyzed individual plot records.

In CV1, our prediction correlations are considerably lower than those of Burgueño et al. (2012) and the differences between the two studies are likely affected by two factors: (a) as mentioned the trait analyzed in Burgueño et al. (2012) is the average of two replicates and therefore it has a higher heritability than the single-plot record used in this study and (b) the strength of genetic relationships among the lines in our study is much weaker, because lines in our data set come from different commercial breeding programs, while in Burgueño et al. (2012) lines come from highly connected breeding programs. In CV1 no records from the own line are available for model fitting; therefore all the prediction accuracy of the models that do not account for G × E comes from borrowing of information among lines, and this is highly affected by genetic relationships. Because the lines used in our study are not as tightly related as those used in Burgueño et al., our baseline correlation is lower (0.175); however, the gains in prediction accuracy obtained by modeling G × E in our study and that of Burgueño et al. (2012) are very similar.

Although ECs and their interactions with markers can explain a sizable proportion of GY variance, we found that the ECs we used in this study explained only a limited proportion of across-environment variation. We also found that even after including ECs and their interaction with markers, a substantial proportion of phenotypic differences were explained by the main effects of environment not accounted for by ECs. Consequently, our most comprehensive model includes—in addition to the effects of markers and ECs and their interaction—the effects of environments and the interactions between markers and environments, whose role in the model was to capture signals that are not captured by markers or ECs. This also suggests that there are opportunities for improving the methods presented here by either considering more ECs or by introducing the ECs in the model in ways that are different from the ones considered here.

We developed our models within the context of a multivariate normal distribution. These models are easy to implemented using existing software for mixed models with structured covariance matrices. However, although the complete model EGW-G × WG × E yielded sizable gains in predictive correlation for CV2 and CV1, our approach is not free of limitations. The Gaussian prior does not induce variable selection and the type of shrinkage induced by the Gaussian prior density may not be appropriate in the presence of large-effect QTLs or large-effect ECs. Therefore, an area of further research would be to extend the methods discussed here to models that induce either differential shrinkage of estimates of effects or a combination of variable selection and shrinkage.

Finally, the models we proposed here considered only one possible mode of interaction: the multiplicative reaction norm model. In practice, interactions between genes and environmental conditions may take many different forms, and the methods proposed here can be considered, at best, a good approximation. Further research on alternative ways of modeling interactions between markers and ECs is warranted.

## Conclusions

Complex traits are affected by large numbers of, possibly interacting, genetic and environmental factors. The continued development of genotyping and sequencing technologies as well as that of information systems that can capture very detailed environmental information opens enormous opportunities for modeling G × E. However, when the number of genetic markers and of ECs is large, modeling all possible interactions between these two sets of variables becomes infeasible. To circumvent this problem we proposed a variance components approach where different covariance structures are used to account for and exploit signals generated by main and interaction effects. The G × E component of the proposed model can be viewed as a reaction norm where the environmental and genetic gradients are modeled as regressions on markers and on ECs. Importantly, the implementation of the proposed model in a REML or Bayesian framework is straightforward.

When the model was used to analyze data from 139 lines evaluated in 340 environments we found that a sizable proportion of the phenotypic variance can be attributed to marker by EC interactions, and that the ability of the model to predict yet-to-be-observed phenotypes increased significantly. These results suggest that the proposed model can be useful for breeding as well as for providing agronomic recommendations tailored to specific environmental conditions. However, the proposed model is not free of limitations and we have outlined research areas that may be pursued to further improve our ability to extract the most out of the wealth of data that is at our disposal.
